# Cardiac ion channel expression in the equine model ‐ In‐silico prediction utilising RNA sequencing data from mixed tissue samples

**DOI:** 10.14814/phy2.15273

**Published:** 2022-07-26

**Authors:** Antoine Premont, Khalil Saadeh, Charlotte Edling, Rebecca Lewis, Celia M. Marr, Kamalan Jeevaratnam

**Affiliations:** ^1^ 3660 Faculty of Health and Medical Sciences University of Surrey Guildford Surrey UK; ^2^ School of Clinical Medicine University of Cambridge Cambridge UK; ^3^ 538458 Rossdales Equine Hospital and Diagnostic Centre Exning Suffolk UK

**Keywords:** calcium‐handling proteins, cardiac arrhythmia, cardiomyocyte, equine cardiac electrophysiology, in‐silico prediction, ion channels, sodium channel, transcriptomics

## Abstract

Understanding cardiomyocyte ion channel expression is crucial to understanding normal cardiac electrophysiology and underlying mechanisms of cardiac pathologies particularly arrhythmias. Hitherto, equine cardiac ion channel expression has rarely been investigated. Therefore, we aim to predict equine cardiac ion channel gene expression. Raw RNAseq data from normal horses from 9 datasets was retrieved from ArrayExpress and European Nucleotide Archive and reanalysed. The normalised (FPKM) read counts for a gene in a mix of tissue were hypothesised to be the average of the expected expression in each tissue weighted by the proportion of the tissue in the mix. The cardiac‐specific expression was predicted by estimating the mean expression in each other tissues. To evaluate the performance of the model, predicted gene expression values were compared to the human cardiac gene expression. Cardiac‐specific expression could be predicted for 91 ion channels including most expressed Na^+^ channels, K^+^ channels and Ca^2+^‐handling proteins. These revealed interesting differences from what would be expected based on human studies. These differences included predominance of Na_V_1.4 rather than Na_V_1.5 channel, and RYR1, SERCA1 and CASQ1 rather than RYR2, SERCA2, CASQ2 Ca^2+^‐handling proteins. Differences in channel expression not only implicate potentially different regulatory mechanisms but also pathological mechanisms of arrhythmogenesis.

## Graphical abstract

Understanding cardiomyocyte ion channel expression is crucial to understanding normal cardiac electrophysiology and underlying mechanisms of cardiac pathologies particularly arrhythmias. Differences in channel expression not only implicate potentially different regulatory mechanisms but also pathological mechanisms of arrhythmogenesis.

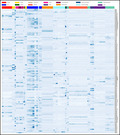

## 1 INTRODUCTION

Electrophysiological properties of excitable cells, including cardiomyocytes, are determined by selective protein expression of surface ion channels regulating ion flux across cellular membranes. In mammals, effective cardiac contraction maintains tissue perfusion, adapts to altered physiological demands such as exercise, and controls arterial blood pressure. These organ‐level functions are dependent upon coordinated cardiomyocyte contraction as determined by the orderly sequence of action potential activation triggering excitation–contraction coupling, propagation, and recovery through successive regions of the myocardium. In turn, this is determined by the activity of sarcolemmal ion channels (Huang, 2017). Thus, disruption to the function or expression of these ion channels will compromise cardiac function and result in a range of possible pathologies including cardiac arrhythmias.

Therefore, knowledge of the ion channels in the heart is crucial to understanding both normal physiology and the underlying mechanisms of cardiac pathologies such as arrhythmias. Furthermore, understanding species‐specific ion channel expression and function will allow targeted use of pharmacological therapies and a better understanding of the cardiac side effects of these therapies (Grant, 2009). For example, despite the relatively high prevalence of atrial fibrillation (AF) in horses with an incidence of up to 2.5% (Decloedt et al., 2020; Else & Holmes, 1971), pharmacological treatment remains limited with only moderate efficacy and significant risk of polymorphic ventricular tachycardia and sudden death (McGurrin, 2015) due to poor understanding of equine electrophysiology and channelome.

Ion channel expression pattern in cardiac tissue has been elucidated in humans and multiple animal models revealing valuable insights on normal physiology (Johnson et al., 2018; Uosaki & Taguchi, 2016) and pathological mechanisms (Lipovsky et al., 2020; Cardin et al., 2008). Despite the clinical importance of cardiac conditions especially in equine athletes in terms of performance and safety issues (Reef et al., 2014), cardiac gene expression investigations have been very limited in the horse. They mostly relied on quantitative polymerase chain reaction (qPCR), western blotting, and immunofluorescence rather than high throughput RNA sequencing technology (Haugaard et al., 2015; Hesselkilde et al., 2014; Pedersen et al., 2015; Pedersen et al., 2017).

“Omics” technologies (genomics, transcriptomics, proteomics, and metabolomics) refer to collective and high‐throughput analyses of biological samples in a non‐targeted and non‐biased manner, allowing complex systems to be understood more thoroughly and, hence, has transformed our understanding of biological processes (Aardema & MacGregor, 2002; Dos Santos et al., 2016). During the last 20 years, they have been extensively applied to both laboratory and clinical research, producing a colossal amount of data on humans, animal models, and pathogens. However, despite a large decrease in the cost of such techniques, their use in veterinary medicine, and particularly in equine medicine, is still limited by funding. Up to now, equine tissue‐specific gene expression data is available for normal brain and muscle but not for the heart. Only one study sequenced a mix of tissues including cardiac tissue (Hestand et al., 2015). Those techniques are precise and sensitive but require a priori determination of a list of target genes. Information on the relative expression of each ion channel in the cardiac channelome can be used to determine the potentially most relevant genes to target.

Therefore, our study aims to predict equine cardiac ion channel gene expression based on open‐source RNA sequencing data obtained from a multi‐tissue mix. Results will guide future directed molecular investigations that will elucidate equine electrophysiology, underlying disease mechanisms, and identify novel therapeutic pharmacological targets.

## 2 MATERIALS AND METHODS

### 2.1 Systematic search strategy

A systematic search for high‐throughput RNA sequencing data from equine tissue was performed on 26 June, 2020 in open‐access databases ArrayExpress and GEO DataSets. The search terms “Equus caballus” were used in both databases.

### 2.2 Data selection and extraction

Dataset was composed of RNA sequencing data of coding or total RNA for which the raw data was available and that included *Equus caballus* as one of the organisms were selected. Raw data (FASTQ files) from normal horses from 9 different datasets was retrieved and reanalysed. One dataset included RNA sequences of a mix of 43 tissues including cardiac muscle. The 8 others included samples from 1 to 6 different tissues. When combined, the 8 datasets included RNA sequences from 12 different tissues present in the mix (skeletal muscle, testis, brain, liver, kidney, cartilage, synovial membrane, cerebellum, embryo, placenta, uterus, and bone) [see Table 1].

**TABLE 1:** RNA sequencing datasets used in the analysis

Reference		Title	Tissue
Roller et al. (2021)	E‐MTAB‐8122	RNA‐seq in 4 tissues of 10 mammals	Muscle, Testis, Liver, Brain
Roller et al. (2021)	E‐MTAB‐8118	RNA‐seq of healthy brain, liver and muscle in 5 mammals	Brain
Fushan et al. (2015)	E‐GEOD‐43013	Gene Expression Defines Natural Changes in Mammalian Lifespan	Liver, Kidney, Brain
Hestand et al. (2015)	E‐MTAB‐2879	Annotation of the Protein Coding Regions of the Equine Genome	43 tissues (Adipose Tissue, Adrenal Cortex, Adrenal Medulla, Aorta, Articular Cartilage (foal), Bladder, Bone, Bone Marrow, Cecum, Cerebrum, Cornea, Embryo (whole embryo, 34d), Endometrium (pregnant day 16), Endometrium (pregnant day 50), Epididymus, Hoof (germinal epithelium), Kidney, Large Intestine, Liver, Lung Lymph Node, Lymphocytes (activated), Muscle (cardiac), Muscle (skeletal, tongue), Muscle (skeletal), Muscle (smooth), Ovary, Pancreas, Pituitary (anterior), Pituitary (posterior), Placental Villous, Retina, Salivary Gland, Skin (full thickness), Spinal Cord, Spinal Root Ganglia, Spleen (red pulp), Spleen (white pulp), Stomach, Synovial Membrane, Tendon (superficial digital flexor, foal), Testes, Thymus, and Vena Cava)
Coleman et al. (2010, 2013)	E‐GEOD‐21925	Structural annotation of equine protein‐coding genes determined by mRNA sequencing	Cartilage, Synovial membrane, Testis, Cerebellum, Embryo, Placenta
Ropka‐Molik et al. (2017)	E‐GEOD‐88951	Exercise‐induced modification of skeletal muscle transcriptome in Arabian horses	Muscle
Scaravaggi et al. (2019)	E‐GEOD‐112237	Endometrial transcriptome changes in comparison of equine endometrium samples collected on Day 12 of pregnancy and Day 12 of the estrous cycle	Uterus
Wang et al. (2013)	E‐GEOD‐30243	Profiling of differential allelic expression in horse, donkey, mule and hinny placental tissue	Placenta
Kemper et al. (2019)	E‐GEOD‐135322	Differential gene expression in articular cartilage and subchondral bone of neonatal and adult horses	Cartilage, Bone

### 2.3 Data processing and analysis

Data was processed and analysed in R‐Studio using the Bioconductor packages *RSubread*, *ShortRead*. The read quality was controlled. Reads were aligned to the equine genome with the function *align* of the *RSubread* package and the number of reads for each gene was counted with the function *FeatureCounts* of the *RSubread* package. Genes were filtered based on their number of reads. Normalisation was performed using the FPKM method. Genes coding ion channels and some ion transporters were selected and a heatmap was generated. The mean FPKM value of each gene was calculated for each tissue and for the mix. To predict the cardiac‐specific gene expression, it was hypothesised that in this mix, the contribution of each tissue to the gene expression only depends on the specific expression level in each tissue and the proportion of each tissue. The normalised (FPKM) read counts *G_ik_*, for a gene *i*, in a sample *k*, is the average of the expected expression in the tissue *j α_ij_* weighted by the proportion of the tissue in the mix *p* supposed to be identical for each tissue (*p* = 1/43) (Equation 1).

(1) $$G_{\mathrm{ik}}=\sum_{j}^{n}\left(\alpha_{\mathrm{ij}}p\right)+\varepsilon_{\mathrm{ik}}$$

The mean cardiac‐specific expression $\bar{H}$ was calculated by estimating the mean expression in each other tissues $\bar{\alpha_{\mathrm{ij}}}$ (Equation 2). When the data was unavailable, the median of the known $\bar{\alpha_{\mathrm{ij}}}$ was used. It was the case for 5 tissues (aorta, cornea, endometrium at 50 days of pregnancy, tendon and vena cava).

(2) $$\bar{H}=\frac{\bar{G_{i}}}{p}‐\sum_{j}^{n‐1}\left(\bar{\alpha_{\mathrm{ij}}}\right)+\varepsilon_{i}$$

For low‐expressed genes, the error term of the model might be higher than the actual expression and resulted in negative expression values. Those genes were removed from the analysis. The standard deviation of the cardiac expression FPKM value was estimated for each gene based on the standard deviation in every tissue.

To evaluate the performance of the model, predicted values of expression of 91 ion channel genes were compared to the expression in human cardiac tissue determined by RNA sequencing as part of the human protein atlas project (Lindskog et al., 2015). When possible, the correlation with qPCR expression data in humans (Gaborit et al., 2007) was also investigated.

## 3 RESULTS

**FIGURE 1:**
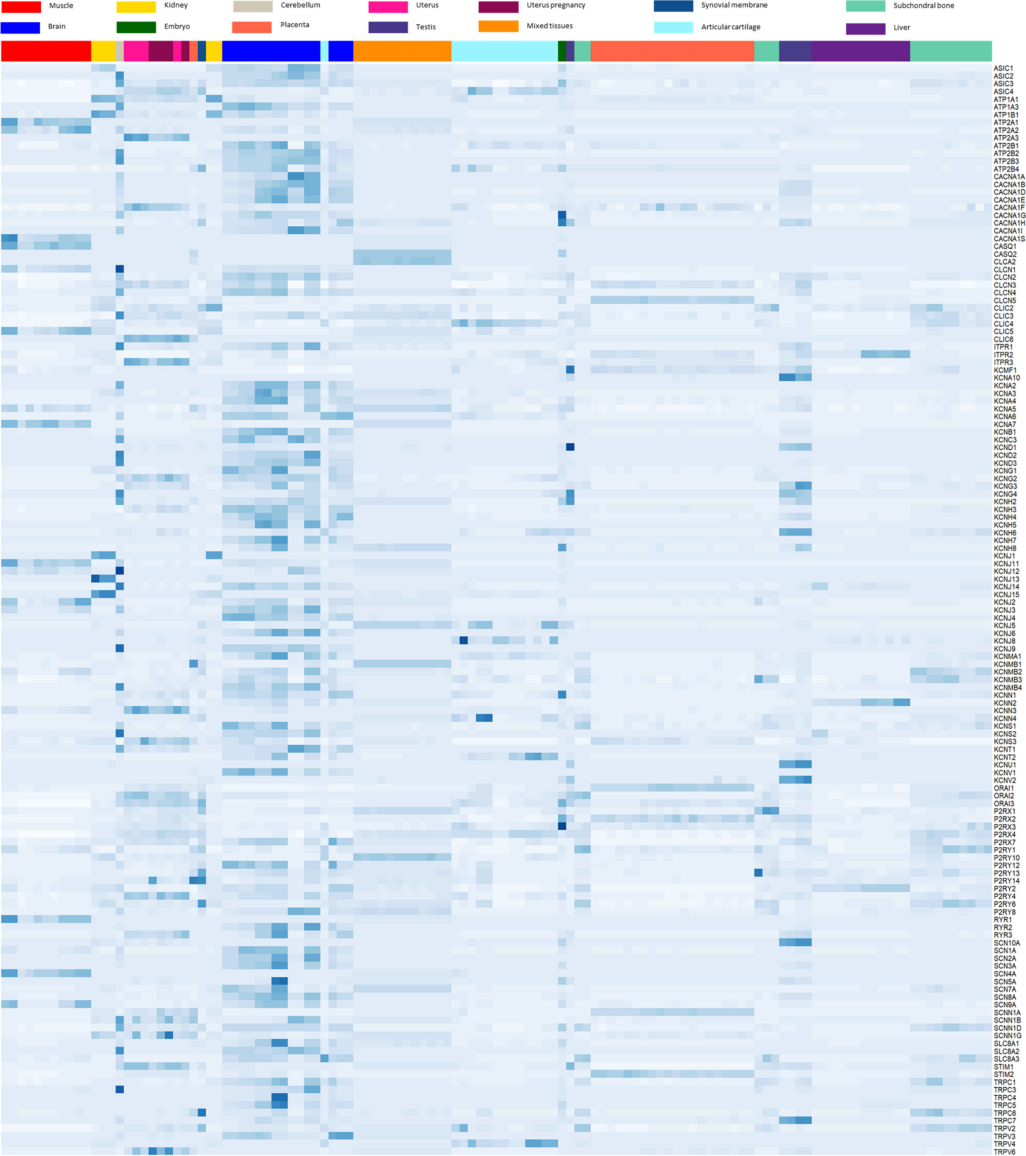
RNA sequencing analysis of ion channel expression in various equine tissues. After filtration, normalization (FPKM), and scaling, a heatmap was constructed and samples were hierarchically clustered

The evaluation of ion channel expression in multiple tissues revealed, as expected, very different tissue‐specific expression patterns [see Figure 1]. Most samples clustered according to their tissue. The uterus samples coming from pregnant and non‐pregnant mares clustered together possibly showing only small differences in ion channel expression related to pregnancy. Samples originating from cerebral tissue showed the highest and the most diversed expression of ion channels. This can be explained by the functional diversity of neurones but also by the fact that the data on brain gene expression is originating from 3 different datasets obtained by 2 different teams. Other tissues with high ion channel expression were muscle, uterus, kidney, and testis. In the multi‐tissue samples, ion channels were lowly expressed but for some of them, the expression was higher than in any other tissue.

The expression of those channels is therefore expected to be high in some of the tissues for which no equine data was available. Therefore, this group of ion channels includes the cardiac‐specific channels.

**FIGURE 2:**
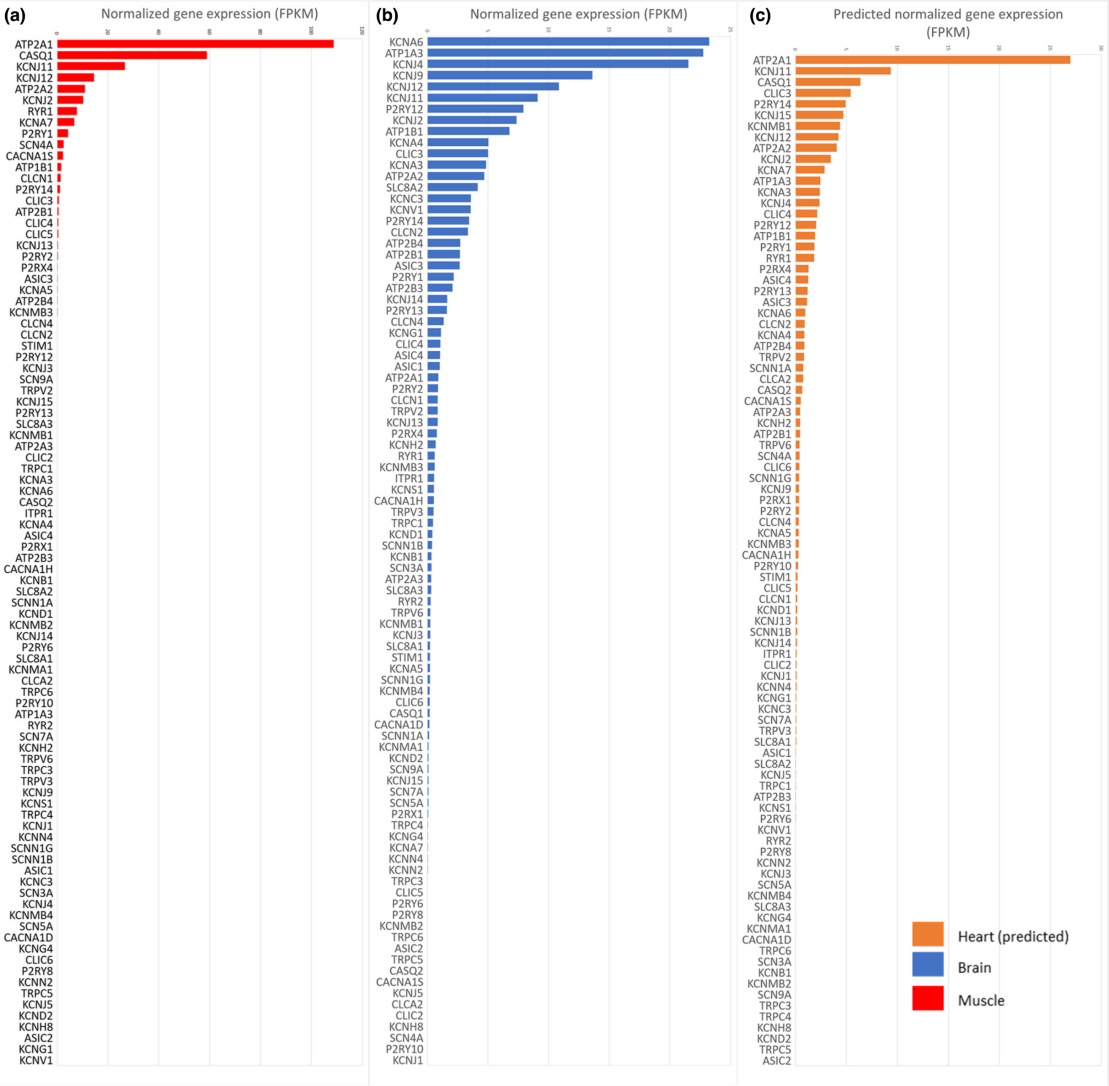
Mean ion channel gene expression in equine excitable tissues. (a) Mean filtered, normalised (FPKM) ion channels expression in equine skeletal muscle. (b) Mean filtered, normalised (FPKM) ion channels expression in equine brain. (c) Predicted ion channels expression in equine cardiac muscle

Cardiac‐specific expression could be predicted for 91 ion channel genes [see Figure 2] including the voltage‐gated sodium channels, delayed‐rectifier, and inward‐rectifying potassium channels as well as some calcium‐handling proteins, pumps, and exchangers. The expression values of the most functionally significant genes are presented in Tables 2 and 3. The voltage‐gated calcium channel Cav1.2 implicated in most of calcium influx in human and mice cardiomyocyte could not be predicted based on the sequencing data because of a too low expression in the multi‐tissue mix.

**TABLE 2:** Gene expression levels of calcium channels, sodium channels, sodium/Calcium exchangers, sodium and calcium pumps, and calcium handling proteins in equine excitable tissue. Data is presented as mean (±standard deviation)

Subunit	Gene	Brain	Muscle	Mix	Heart (predicted)
A. Calcium channels					
Cav1.1	CACNA1S	0.014 (0.006)	2.333 (1.424)	0.580 (0.011)	0.521 (0.430)
Cav1.3	CACNA1D	0.185 (0.095)	0.001 (0.001)	0.019 (0.001)	0.007 (0.030)
Cav3.2	CACNA1H	0.544 (0.280)	0.020 (0.006)	0.348 (0.010)	0.258 (0.108)
B. Sodium channels					
Nav1.3	SCN3A	0.346 (0.223)	0.001 (0.001)	0.021 (0.002)	0.010 (0.064)
Nav1.4	SCN4A	0.002 (0.002)	2.617 (1.049)	0.481 (0.021)	0.393 (0.326)
Nav1.5	SCN5A	0.097 (0.190)	0.001 (0.001)	0.028 (0.002)	0.015 (0.050)
Nav1.7	SCN7A	0.097 (0.083)	0.005 (0.005)	0.102 (0.005)	0.093 (0.023)
Nav1.9	SCN9A	0.107 (0.076)	0.109 (0.105)	0.013 (0.001)	0.007 (0.037)
C. Sodium/calcium exchangers					
NCX1	SLC8A1	0.227 (0.214)	0.011 (0.001)	0.093 (0.002)	0.076 (0.056)
NCX2	SLC8A2	4.160 (1.508)	0.016 (0.007)	0.195 (0.012)	0.047 (0.390)
NCX3	SLC8A3	0.320 (0.132)	0.071 (0.047)	0.039 (0.002)	0.015 (0.087)
D. Sodium and calcium pumps					
Na/K ATPase α3	ATP1A3	22.77 (9.534)	0.006 (0.005)	3.061 (0.094)	2.441 (2.485)
Na/K ATPase β1	ATP1B1	6.771 (4.390)	1.625 (0.712)	3.043 (0.059)	0.812 (1.319)
PMCA1	ATP2B1	2.702 (1.442)	0.594 (0.427)	0.676 (0.017)	0.003 (0.426)
PMCA3	ATP2B3	2.077 (0.756)	0.025 (0.014)	0.125 (0.006)	0.051 (0.136)
PMCA4	ATP2B4	2.724 (1.235)	0.277 (0.155)	1.148 (0.020)	0.139 (0.476)
SERCA1	ATP2A1	0.919 (0.119)	108.9 (41.96)	29.80 (0.473)	26.00 (12.66)
SERCA2	ATP2A2	4.688 (1.994)	10.94 (5.918)	4.87 (0.114)	1.934 (2.006)
SERCA3	ATP2A3	0.329 (0.115)	0.051 (0.017)	3.463 (0.042)	0.122 (3.842)
E. Calcium handling					
CASQ1	CASQ1	0.197 (0.130)	59.02 (16.23)	7.794 (0.264)	6.330 (4.900)
CASQ2	CASQ2	0.016 (0.024)	0.0350 (0.020)	0.682 (0.017)	0.672 (0.024)
RYR1	RYR1	0.624 (0.250)	7.900 (4.188)	2.043 (0.041)	1.804 (1.265)
RYR2	RYR2	0.281 (0.198)	0.005 (0.004)	0.035 (0.001)	0.022 (0.053)

**TABLE 3:** Gene expression levels of potassium channel proteins in equine excitable tissue. Data is presented as mean (± standard deviation)

Subunit	Gene	Brain	Muscle	Mix	Heart (predicted)
A. Delayed rectifier K^+^ channels					
Kv1.3	KCNA3	4.842 (2.462)	0.040 (0.027)	2.620 (0.139)	1.944 (0.747)
Kv1.5	KCNA5	0.224 (0.123)	0.285 (0.107)	0.343 (0.024)	0.268 (0.068)
Kv1.6	KCNA6	23.25 (10.67)	0.037 (0.026)	1.731 (0.124)	0.767 (3.494)
Kv1.7	KCNA7	0.064 (0.040)	6.804 (1.764)	3.053 (0.149)	2.842 (0.537)
Kv2.1	KCNB1	0.349 (0.141)	0.019 (0.010)	0.020 (0.002)	0.003 (0.037)
B. A‐type K^+^ channels					
Kv1.4	KCNA4	5.052 (2.654)	0.032 (0.026)	1.074 (0.085)	0.766 (0.762)
Kv3.3	KCNC3	3.601 (2.116)	0.002 (0.002)	0.208 (0.015)	0.082 (0.549)
Kv4.1	KCND1	0.421 (0.201)	0.014 (0.010)	0.327 (0.032)	0.116 (1.154)
Kv4.2	KCND2	0.111 (0.077)	0.000 (0.000)	0.004 (0.005)	0.001 (0.020)
C. Inward rectifying K^+^ channels					
Kv11.1/ERG	KCNH2	0.704 (0.318)	0.005 (0.001)	0.548 (0.022)	0.455 (0.367)
D. Slowly activating K^+^ channels					
Kv12.1	KCNH8	0.003 (0.003)	0.000 (0.000)	0.005 (0.001)	0.004 (0.002)
E. Inward rectifying KIR channels					
Kir1.1	KCNJ1	0.001 (0.003)	0.003 (0.003)	0.729 (0.058)	0.117 (0.776)
Kir4.2	KCNJ15	0.103 (0.160)	0.100 (0.103)	7.634 (0.417)	3.474 (26.89)
Kir7.1	KCNJ13	0.859 (0.872)	0.450 (0.436)	0.972 (0.066)	0.007 (10.13)
Kir2.1	KCNJ2	7.372 (3.150)	10.34 (7.724)	4.062 (0.236)	2.634 (2.568)
Kir2.2	KCNJ12	10.87 (12.89)	14.64 (7.135)	4.897 (0.199)	4.080 (3.976)
Kir2.3	KCNJ4	21.53 (9.594)	0.001 (0.003)	2.899 (0.241)	2.350 (2.480)
Kir2.4	KCNJ14	1.645 (0.508)	0.013 (0.007)	0.249 (0.024)	0.019 (0.215)
F. G‐protein‐gated K^+^ channels					
Kir3.1	KCNJ3	0.247 (0.124)	0.109 (0.081)	0.034 (0.002)	0.022 (0.041)
Kir3.3	KCNJ9	13.61 (4.386)	0.004 (0.008)	0.716 (0.082)	0.349 (1.143)
Kir3.4	KCNJ5	0.014 (0.009)	0.000 (0.000)	0.086 (0.005)	0.078 (0.016)
G. ATP‐sensitive K^+^ channels					
Kir6.2	KCNJ11	9.087 (3.240)	26.70 (10.85)	10.30 (0.340)	8.992 (3.555)
H. Modifier/silencer K^+^ channels					
Kv6.1	KCNG1	1.134 (0.730)	0.000 (0.000)	0.155 (0.017)	0.106 (0.198)
Kv6.4	KCNG4	0.066 (0.073)	0.001 (0.001)	0.032 (0.005)	0.014 (0.065)

The model predicted higher FPKM values for RYR1, CASQ1, and SERCA1 compared to RYR2, CASQ2, and SERCA2, respectively. SCN4A had a higher predicted value than SCN5A or SCN7A. The most expressed rectifier K^+^‐channels according to the prediction were KCNA7, KCNA3, KCNA4, KCNA6, and KCNH2. The KIR K^+^‐channels responsible for the I_K1_ current determining the resting membrane potential showed very high variability and poor confidence in predicted values. Those channels are more ubiquitously expressed across tissues which make their cardiac expression prediction less reliable. Predicted expression values for KCNJ11 responsible for the ATP‐sensitive K^+^‐current were high.

**FIGURE 3:**
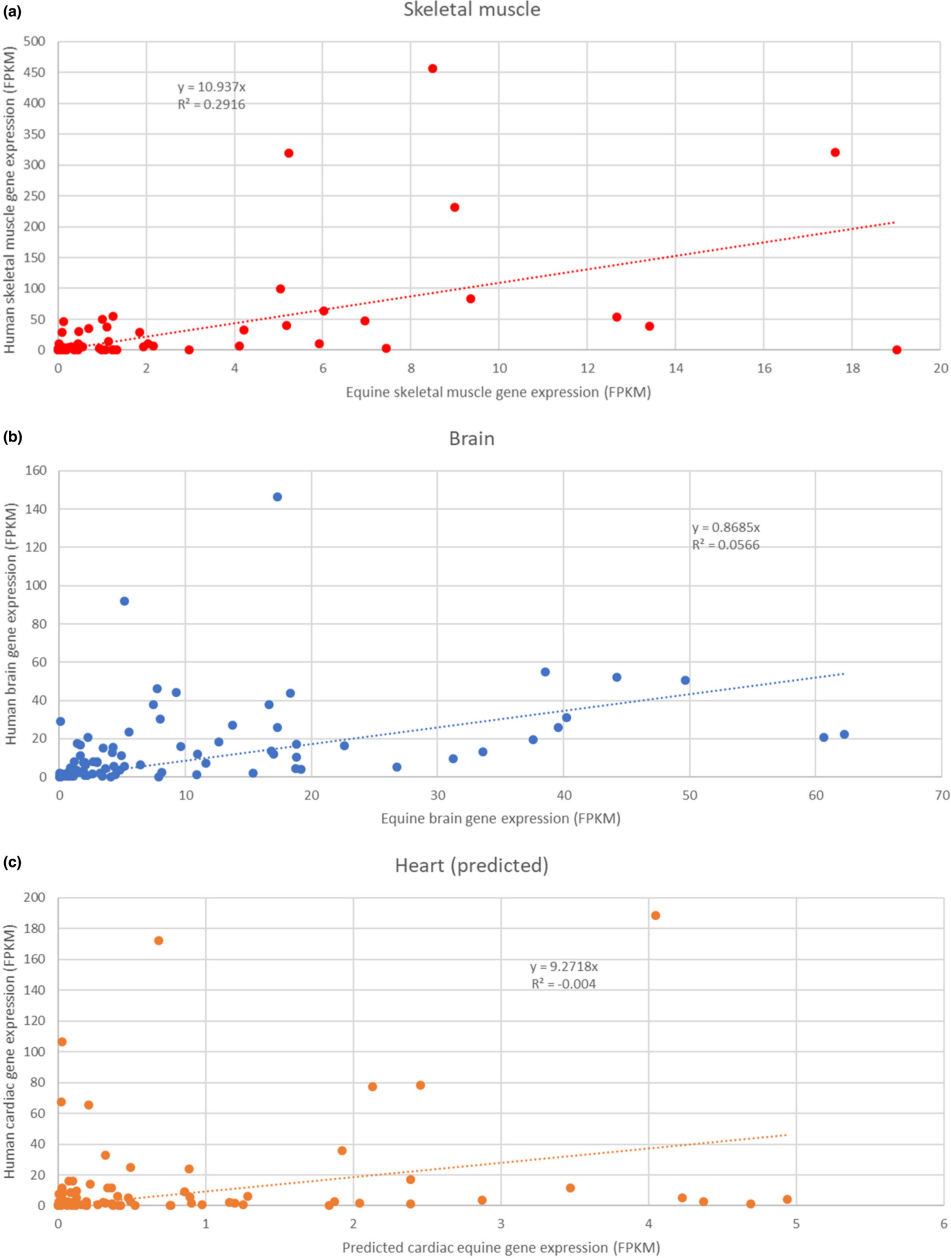
Correlation between human and equine ion channel gene expression in excitable tissues. (a) Correlation between equine and human RNA sequencing skeletal muscle ion channel expression data. A significant correlation was observed with a Pearson’s correlation test (coefficient = 0.545, *T*‐statistic = 6.127, *df* = 89, *p* < 0.0001). (b) Correlation between equine and human RNA sequencing brain ion channel expression data. A significant correlation was observed with a Pearson’s correlation test (coefficient = 0.387, *T*‐statistic = 3.965, *df* = 89, *p* < 0.001). (c) Correlation between equine (predicted) and human RNA sequencing cardiac ion channel expression data. After removing 4 outliers, a significant correlation was observed with a Pearson’s correlation test (coefficient = 0.478, *T*‐statistic = 4.809, *df* = 78, *p* < 0.001)

**FIGURE 4:**
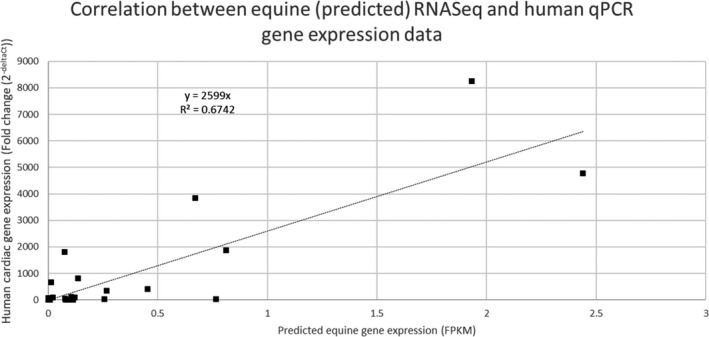
Correlation between equine (predicted) RNA sequencing and human qPCR cardiac ion channel expression data. A significant correlation was observed with a Pearson’s correlation test (coefficient = 0.821, *T*‐statistic = 6.756, *df* = 22, *p* < 0.001)

Comparison with human data for the 91 ion channels showed a shift towards lower FPKM values for equine gene expression in muscle tissue as well as for predicted values in the heart. This may be explained by the difference in sequencing depth between the human and the equine studies or species‐specific differences in gene expression pattern. However, the human expression values showed a statistically significant correlation with the experimental equine values in brain and muscle tissue (Pearson’s correlation test, *p* < 0.001) but not with the predicted values. The precision of the prediction was low, and the confidence interval was large for some expression values. As correlation analysis can be highly influenced by outliers, the imprecision on the genes with the highest expression values had great influence on the correlation coefficient. To limit this risk, the most extreme outliers defined as 3 interquartile range above the 3rd quartile were removed. It is expected for the distribution of gene expression values to be skewed in any tissue with a relatively small number of genes very highly expressed and a great majority with much lower expression value. Therefore, the approach chosen to identify and remove potential outliers was intentionally very conservative. Removing the 4 outliers was enough to achieve a statistically significant correlation (coefficient = 0.218, *T*‐statistic = 2.059, *df* = 85, *p* < 0.05) [see Figure 3]. However, the Pearson correlation coefficients are rather low with values of 0.545, 0.387, and 0.218 for brain, muscle, and cardiac tissue, respectively. A statistically significant correlation between the predicted equine RNA sequencing and the experimental human qPCR cardiac ion channel expression data was observed (Pearson’s correlation test, coefficient = 0.821, *T*‐statistic = 6.756, *df* = 22, *p* < 0.001) [see Figure 4].

## 4 DISCUSSION

This study is the first to investigate the whole cardiac equine channelome. It provides a predicted image of the whole channelome of the equine heart at the transcriptomic level. Despite molecular studies previously characterising ion channel expression in other mammalian species, presumed similarity of gene expression data from one species to another is highly unreliable. This is particularly the case for cardiac tissue. This is problematic for both human and veterinary medicine. Horses have long been used to study cardiac diseases being used as a model for a variety of conditions affecting humans such as AF (Carstensen et al., 2019; Hesselkilde et al., 2017; Hesselkilde et al., 2019; Van Loon et al., 2002) but also to model healthy electrophysiological adaptations (Li et al., 2018). However, without fully understanding the equine channelome, the utility of the equine model to investigate human conditions is significantly limited. Additionally, the equine species have evolved through natural and human selection towards high aerobic capacity based on extremely efficient musculoskeletal and cardio‐respiratory systems (Poole & Erickson, 2008; Poole & Erickson, 2011). The availability of such equine‐specific data for those tissues is therefore essential to study the physiological specificity of the horse and identify the factors responsible for exercise performance as well as exercise‐related diseases. Moreover, this paucity of data limits our ability to understand and effectively and safely treat cardiac diseases in horses. For example, AF is the most clinically relevant arrhythmia in horses with a reported incidence of up to 2.5% (Decloedt et al., 2020; Else & Holmes, 1971). However, pharmacological treatment of AF, such as with quinidine, is limited by moderate efficacy and potentially severe side effects including polymorphic ventricular tachycardia and sudden death (McGurrin, 2015). Better knowledge of the equine cardiac channelome will allow a better understanding of these side effects and inevitably will lead to the development of safer and more effective anti‐arrhythmic therapies.

The confidence in the predicted values obtained in this study was unfortunately low. This could be explained by the important variability between the samples included. The sequencing data was obtained by different teams from different horses for different purposes in each study leading to possibly very different gene expression. In addition, some tissues can have very heterogenous cellular composition and therefore gene expression pattern. For example, different brain region (Khaitovich et al., 2004) or different muscle fibres types (Rubenstein et al., 2020) can display different gene expression profile. This is especially the case in the heart where previous studies in other species have demonstrated differing ion channel expression not only between atria and ventricles but also within different regions of each chamber such as between the epicardium and myocardium (Bartos et al., 2015; Saadeh, Chadda, et al., 2020; Schram et al., 2002;). Hence, the method and site of sampling can, for those tissues, explain the differences between samples. The sequencing depth especially of the multi‐tissue mix may have limited our ability to evaluate the lowest expressed and the most tissue‐specific genes. The model used in this study was based on the assumption that in the mix of tissue, the contribution to each tissue linearly depends on the expression level of the gene in the tissue. This assumption is an extension of the linearity assumption in deconvolution methods used to estimate cell composition from bulk RNA sequencing data (Wang et al., 2019). However, the model still relies on the homogeneity of the tissue composition in the mix of tissue so that every tissue contributed equally to the total RNA bulk and this could not be confirmed. Other experimental variation such as sequencing depth, sampling method, or horse population may have influenced the gene expression profile and decreased the accuracy of the prediction.

The similarity of ion channel gene expression in muscle, brain, and cardiac tissue between horses and humans was evaluated using a correlation approach. The rational for this approach is based on previous comparison of ortholog gene expression profiles between human patients and murine model in multiple tissues (Chan et al., 2009; Xing et al., 2007; Zheng‐Bradley et al., 2010). Our study showed limited but significant correlation between equine and human data. The strength of those correlations was similar to those between human and murine samples. Both methodological disparities as discussed above, and biological specificities can explain such difference in gene expression profile. Interspecific variations in gene expression profile, even between closely related species, have been demonstrated in several tissue including brain muscle and heart (Blake et al., 2020; Enard et al., 2002; Roller et al., 2021). Species‐specific differences can be explained by differences in the expression of a small number of genes, but the overall correlation is expected to remain true as the general function of the tissue is the same in all mammalian species.

Interestingly, according to the present study, the predicted most expressed sodium channels, potassium channels, and calcium‐handling proteins differed from what would be expected based on human studies. Voltage‐gated sodium (Na_V_) channels are responsible for the rapid depolarisation phase (phase 0) of the cardiac action potential and hence determines action potential activation and conduction velocity (King et al., 2013). Na_V_1.5, encoded by SCN5A, is the dominant isoform in human and pig heart (Priest & McDermott, 2015; Tanner & Beeton, 2018). Alterations in Na_V_1.5 have been associated with a number of conditions such as loss‐of‐function mutations causing Brugada syndrome, and gain‐of‐function mutations causing long QT Syndrome type 3 (Jeevaratnam et al., 2016; Saadeh, Chadda, et al., 2020). Contrastingly, our results predict that Na_V_1.4, encoded by SCN4A, is the predominant equine cardiac sodium channel highlighting an important difference to human hearts. Different Na_V_ channel isoforms have important structural and functional differences including activation and inactivation kinetics which will have implications on cardiac electrophysiology. When transfected to HEK cells, Nav1.4 channel has a more depolarised inactivation curve and a faster recovery from inactivation compared to Nav1.5. Coupling with Nav1.4 expressing HEK cells increases the maximal depolarisation velocity of cardiomyocyte action potential (Lu et al., 2012). This experimental data suggests that Nav1.4 expression could lead to faster conduction velocity reducing the arrhythmogenic substrate for re‐entry and improve cardiac excitability minimising the risk of conduction blocks. Nevertheless, the higher expression predicted for SCN4A by our model is related to the very high expression value and variability for this sodium channel in muscle tissue that might have altered the prediction for the heart. Further targeted research is required to confirm this prediction and investigate the role of Na_V_1.4 in equine hearts.

A great diversity of potassium channels can be expressed in the heart with varying physiological properties and pathological implications (Antzelevitch, 2004). The normal membrane resting potential is maintained primarily by outward K^+^ currents across inward rectifier K^+^ channels (I_K1_) (Huang, 2017). Following membrane depolarisation, outward membrane current resulting from K^+^ channel opening drives action potential repolarisation, controlling action potential duration whose prolongation is associated with arrhythmic tendency. These include transient outward (I_to_) and delayed rectifier currents (I_Kr_, I_Ks_) (Huang, 2017; Jeevaratnam et al., 2018; Saadeh et al., 2021). It is known that there is significant species‐dependent variety of K^+^ channels. The present prediction indicates that the most expressed rectifier K^+^‐channels were KCNA7, KCNA3, KCNA4, KCNA6 and KCNH2. However, the channels giving rise to the I_K1_ current showed high variability and poor confidence in predicted values. Those channels are more ubiquitously expressed across tissues which makes their cardiac expression prediction less reliable. Nonetheless, the predicted expression values for KCNJ11 responsible for the ATP‐sensitive K^+^‐current were high. This channel is particularly important in the electrophysiological response to stresses such as ischemia (Zhuo et al., 2005). It is also involved in the cardiac adaptation to exercise. In mice, its expression increases with training and facilitates the action potential shortening at high heart rates reducing the cardiac energy consumption (Zingman et al., 2011). In horses, the high expression of this channel could be related to the adaptation of this species to high athletic demands. In addition to inter‐species differences, there is a significant regional heterogeneity in K^+^ channel expression (Barth et al., 2005; Gaborit et al., 2007; Johnson et al., 2018) thus making it difficult to interpret our results without knowing where the cardiac samples were taken from. The predicted expression of KCNA5 responsible for the atrial‐specific I_Kur_ current (Gaborit et al., 2007) was very low suggesting that the equine cardiac sample may have originated from the ventricles. The high expression of KCNA4 responsible for the I_to_ current is more pronounced in the ventricle points to same conclusion. Only KCNH2 responsible for the rapid component of the delayed rectifier current I_Kr_, and not KCNQ1 responsible for I_Ks_ could be predicted. The repolarisation phase of the equine action potential could therefore be different from the human action potential. However previous experiments have shown the expression of KCNQ1 in equine ventricle at the RNA and protein level (Finley et al., 2002). Horses have a lower resting heart rate compared to human but can also reach maximal heart rate over 200 beats per minutes which suggest more drastic changes of the action potential at exercise. The great electrophysiologic compliance of the equine cardiac tissue is an important characteristic that would require specific repolarisation properties (Li et al., 2018). More studies are required to fully characterise the molecular basis for cardiac repolarisation in the horse.

With a 10,000‐fold transmembrane gradient, Ca^2+^ is the most tightly regulated intracellular ion being utilised virtually ubiquitously in cellular signalling pathways but particularly in cardiomyocyte contraction making it central to cardiac function (Bers, 2008). The L‐type voltage‐gated Ca^2+^‐channel Ca_V_1.2 is the dominant channel involved in excitation–contraction coupling being responsible for the majority of the inward current during the plateau phase of the cardiac action potential (Priest & McDermott, 2015). However, Ca_V_1.2 expression could not be predicted due to low expression levels in the multi‐tissue mix. In addition to sarcolemmal Ca^2+^ entry, intracellular Ca^2+^ is regulated by a number of handling proteins. These include ryanodine receptor (RyR) responsible for Ca^2+^ release from the sarcoplasmic reticulum (SR), SR Ca^2+^‐binding calsequestrin (CASQ) which exerts important modulatory inhibitory or enhancing effects on RyR‐mediated Ca^2+^ release at low or high SR luminal Ca^2+^ concentrations, respectively (Chen et al., 2013; Györke & Terentyev, 2008; Handhle et al., 2016), and the cardiac SR Ca^2+^‐ATPase (SERCA) which is key to terminating the cardiac cycle through diastolic reuptake of cytosolic Ca^2+^ into the SR (Bers, 2002). Disruption in any of the Ca^2+^ handling component will inevitably compromise cardiac function and has been associated with a wide range of conditions including proarrhythmic conditions such as catecholaminergic polymorphic ventricular tachycardia (CPVT) (Saadeh, Achercouk, et al., 2020). Of those Ca^2+^ handling proteins, the RYR2, SERCA2, and CASQ2 are the most expressed isoforms in the human heart whereas our model predicted RYR1, SERCA1, and CASQ1 to be the most expressed isoforms in equine hearts. It remains unclear if this surprising difference of isoforms results from a true biological variation or a prediction error due to the highly variable expression in muscle tissue. Nevertheless, different isoforms are known to exhibit different properties. RYR1 is mostly expressed in muscle while RYR2 is cardiac specific. SERCA1 is regulated by sarcolipin whereas SERCA2 is regulated by phospholamban and they have different properties of calcium leakage and ADP‐sensitivity (Schiaffino & Reggiani, 2011). CASQ1 has more calcium‐binding capacity (Beard et al., 2004). RYR1, SERCA1, and CASQ1 are all expressed in fast muscle fibres (Schiaffino & Reggiani, 2011). These 3 isoforms are responsible for fast muscle fibres for higher peak calcium transient. Myosin isoforms of fast muscle fibres allow higher contraction velocity and greater isomeric tension and peak power in response to this calcium kinetics (Schiaffino & Reggiani, 2011). It is possible that this different gene expression profile of cardiac tissue in horses is related to the size and athletic specificity of the horse allowing the equine myocardium to develop more power. Differences in Ca^2+^ handling proteins may result in different patterns of Ca^2+^ homeostasis and hence differences in underlying disease pathologies especially regarding arrhythmic tendency. Cardiac arrhythmia such as premature depolarisation can be triggered by afterdepolarisations due to altered calcium handling. When the amount of calcium released in the cytoplasm overcome the reabsorption capacity of the sarcoplasmic reticulum, calcium expulsion through Na^+^/Ca^2+^‐exchanger creates a depolarising current that can trigger a new action potential (Katz, 2010). Furthermore, elucidating the role of these Ca^2+^ handling proteins may offer potentially novel therapeutic targets. These possibilities are currently being investigated in humans for treatment of arrhythmia and heart failure (Njegic et al., 2020, Gregory and Kranias, 2006).

## CONFLICTS OF INTEREST

The authors declare no conflict of interest. The funders had no role in the design of the study; in the collection, analyses, or interpretation of data; in the writing of the manuscript, or in the decision to publish the results.

## AUTHOR CONTRIBUTIONS

Conceptualization, KJ, RL and AP.; methodology, AP; validation, AP, KS, CEE, KJ; formal analysis, AP, KS; data curation, AP; writing—original draft preparation, AP; writing—review and editing, AP, KS, RL, CEE, KJ; supervision, KJ, RL, CMM; project administration, KJ; funding acquisition, KJ, CMM All authors have read and agreed to the published version of the manuscript.

## ETHICS STATEMENT

The data used in this paper were obtained form previously published studies. The experimental methods in all studies were performed in accordance with the relevant guidelines and regulations.
